# Supplementary figures and images for: All-synchronized picosecond pulses and time-gated detection improve the spatial resolution of two-photon STED microscopy in brain tissue imaging
Source: PLoS One. 2023 Aug 24;18(8):e0290550. doi: 10.1371/journal.pone.0290550 (PMC10449175; doi:10.1371/journal.pone.0290550)

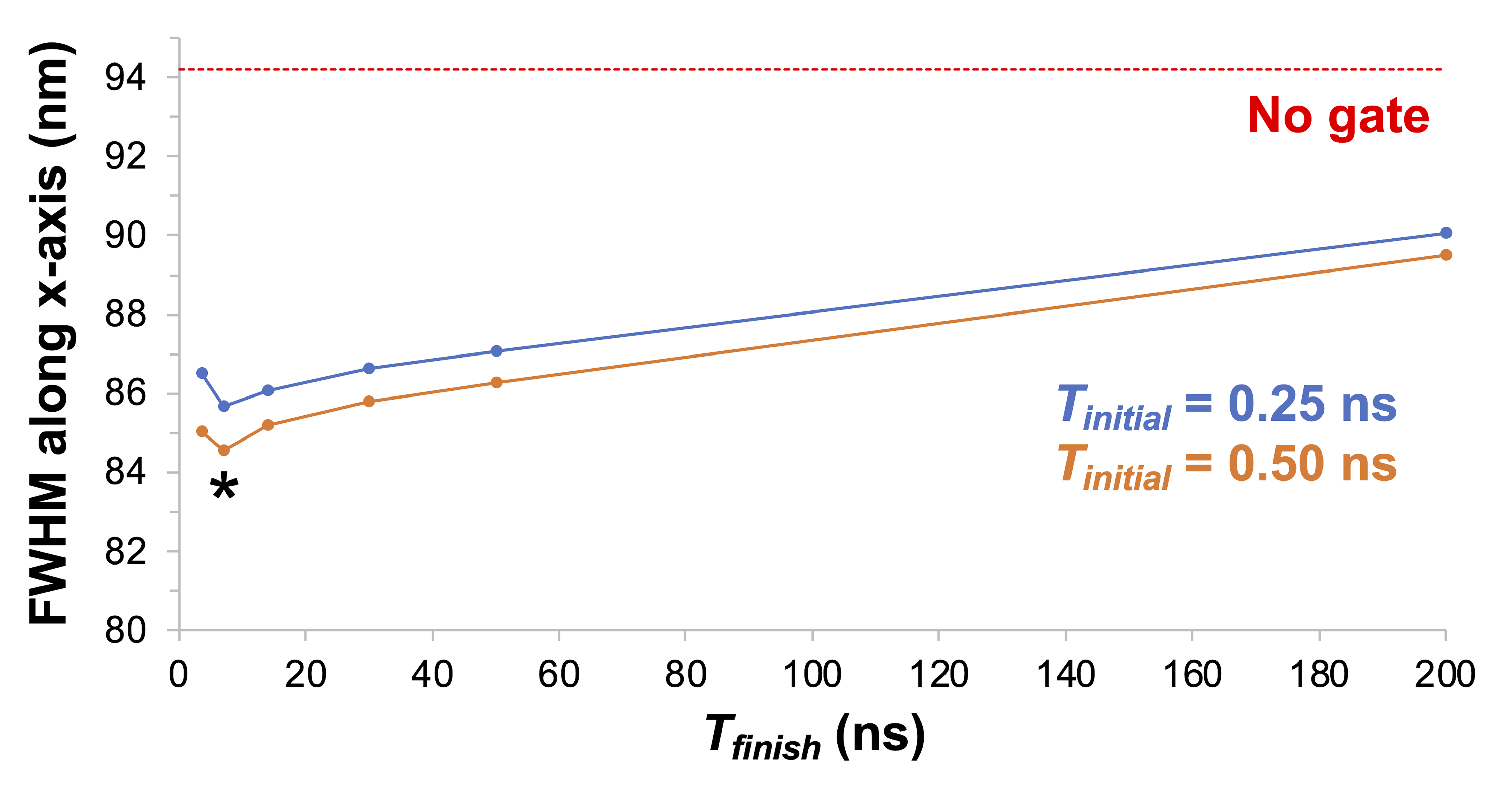

Supplement: S1 Fig — The FWHM values along the x-axis were evaluated within each gating window for the final image of all 41 images of a single Nile red bead from 2PE-STED imaging (analyzed in Fig 2). The dotted red line indicates the FWHM value of the final 2PE-STED image without time-gating (no gate). Tinitial was set to 0.25 ns or 0.50 ns, and Tfinish was set arbitrarily. Setting ΔT to 0.50–7.00 ns gave the best FWHM (asterisk). (TIF) [file pone.0290550.s001.tif]

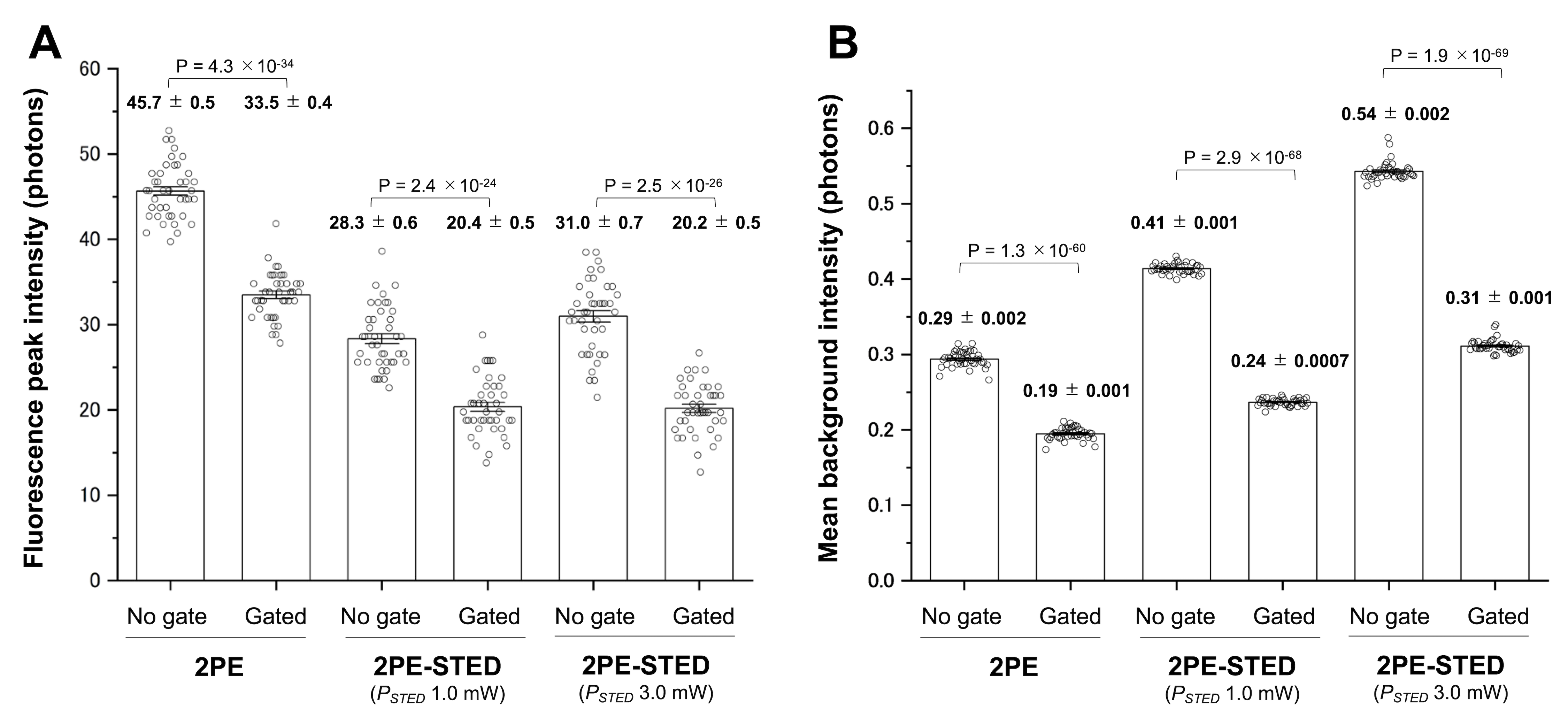

Supplement: S2 Fig — (A) Comparison of the 2PE, 2PE-STED, and gated 2PE-STED (2PE-gSTED) imaging of the 20 nm Nile red bead used in Fig 2. Fluorescence peak intensities of all 41 images of a single Nile red bead under each condition. The error bars represent the s.e.m. The P-values are from a paired Student’s t-test. (B) The same ROI was manually set to the background region of the 2PE, 2PE-STED, and 2PE-gSTED images, and the mean intensities in the ROIs were compared under each condition. The error bars represent the s.e.m. The P-values are from a paired Student’s t-test. (TIF) [file pone.0290550.s002.tif]

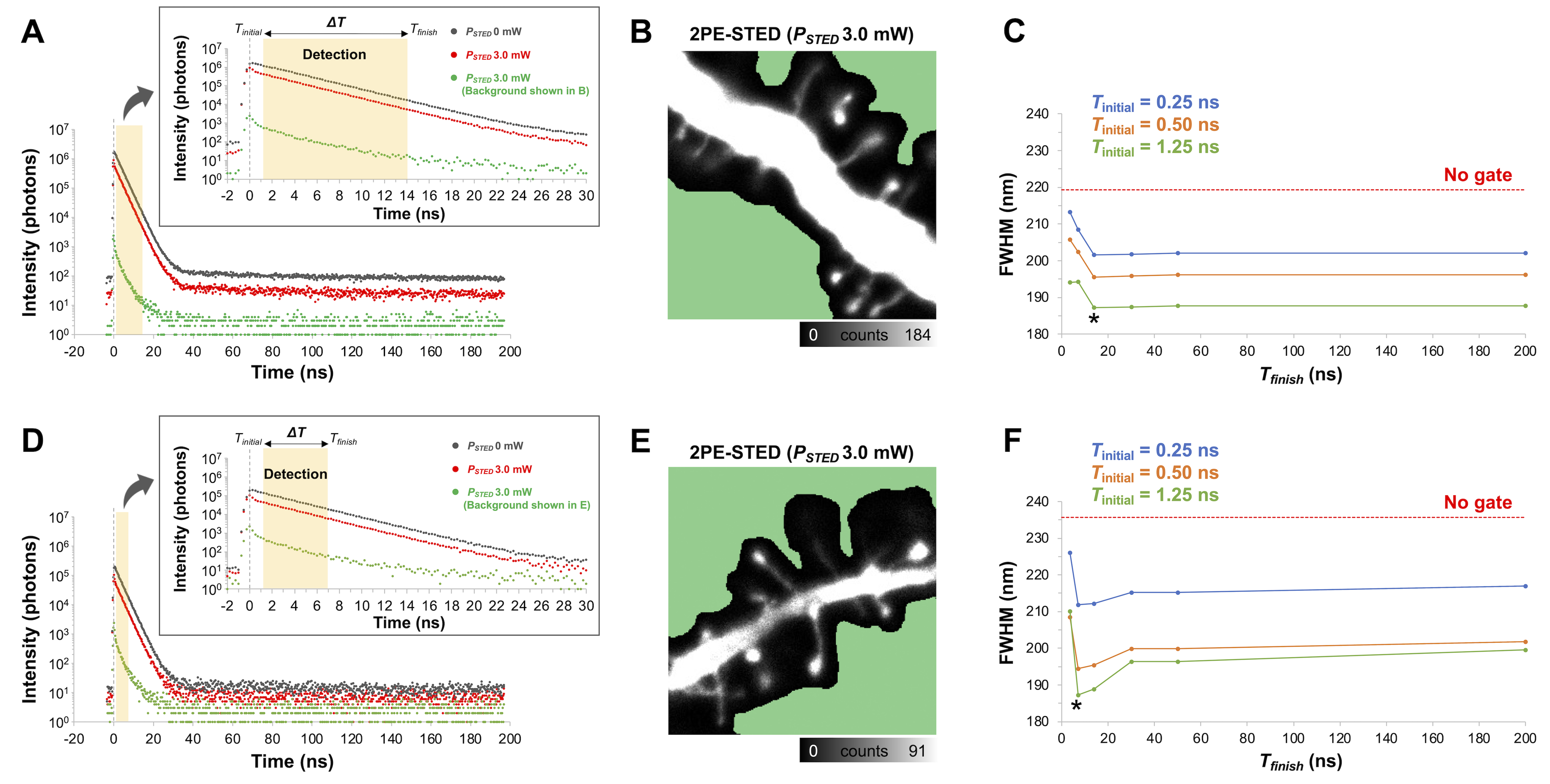

Supplement: S3 Fig — (A, D) Histogram of the photon-arrival times of the fluorescence of the neuronal dendrite observed with 2PE or 2PE-STED microscopy at 4 μm depth (A) and 36 μm depth (D). The constructed images from the data in A and D were shown in Fig 3B and 3F, respectively. The photon-arrival times from the background region of the 2PE-STED images at the 4 μm and 36 μm depths (B and E, respectively) are also plotted on each histogram. (C, F) The FWHM values along the spine necks in the 2PE-STED images at both depths were evaluated within each gating window. The dotted red line indicates the FWHM value without time-gating (no gate). Tinitial was set to 0.25 ns, 0.50 ns, and 1.25 ns. Tfinish was set arbitrarily. The best FWHM value was obtained (asterisks) at ΔT = 1.25–14.00 ns for the 4-μm-depth image and ΔT = 1.25–7.00 ns for the 36-μm-depth image. (TIF) [file pone.0290550.s003.tif]

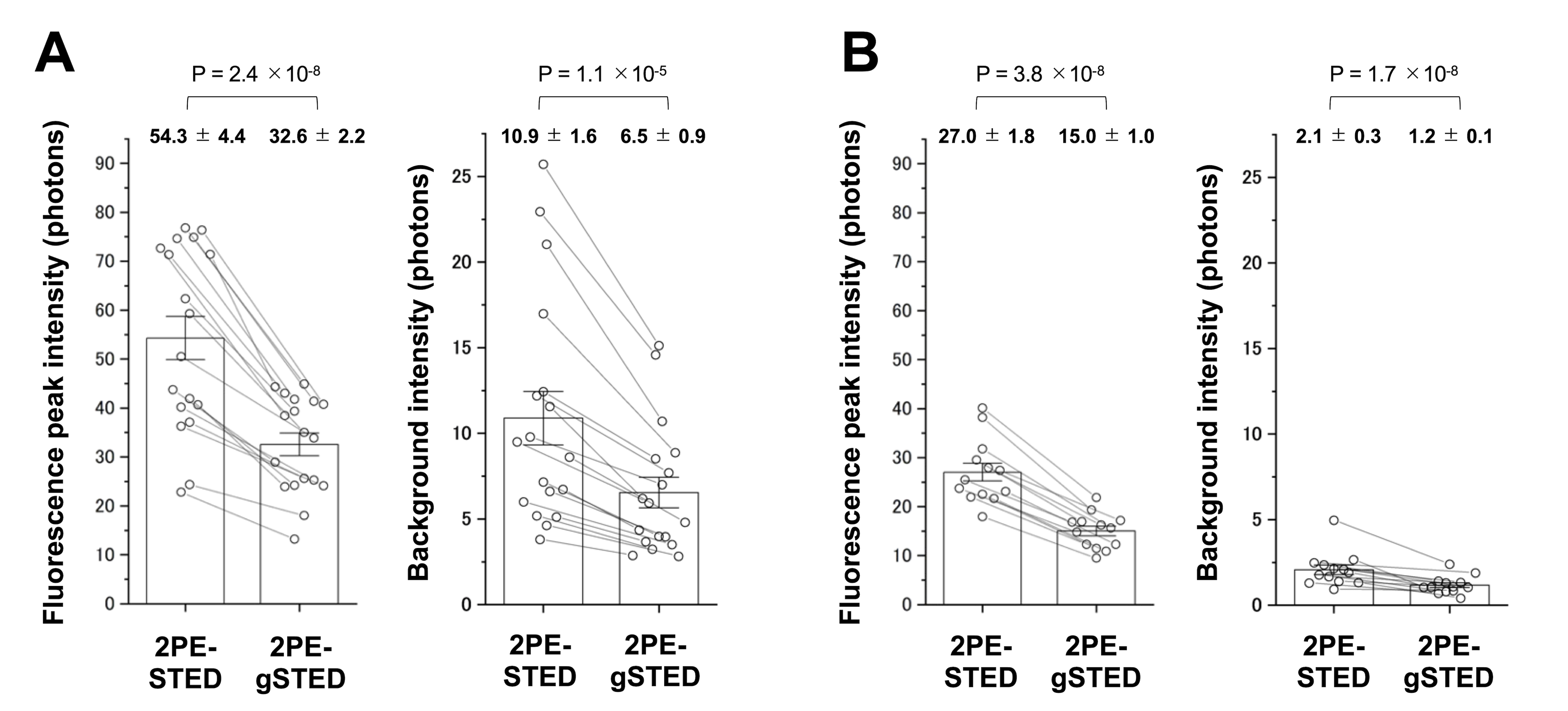

Supplement: S4 Fig — The fluorescence peak and background intensities in the local area around the spine necks in the 2PE-STED and 2PE-gSTED images used in Fig 3 were obtained from the Gaussian curve parameters. The fluorescence peak intensities were obtained from the amplitude parameters, and the background intensities were obtained from the offset parameters at the depths of 4 μm (A) and 36 μm (B). The error bars represent the s.e.m. The P-values are from a paired Student’s t-test. (TIF) [file pone.0290550.s004.tif]

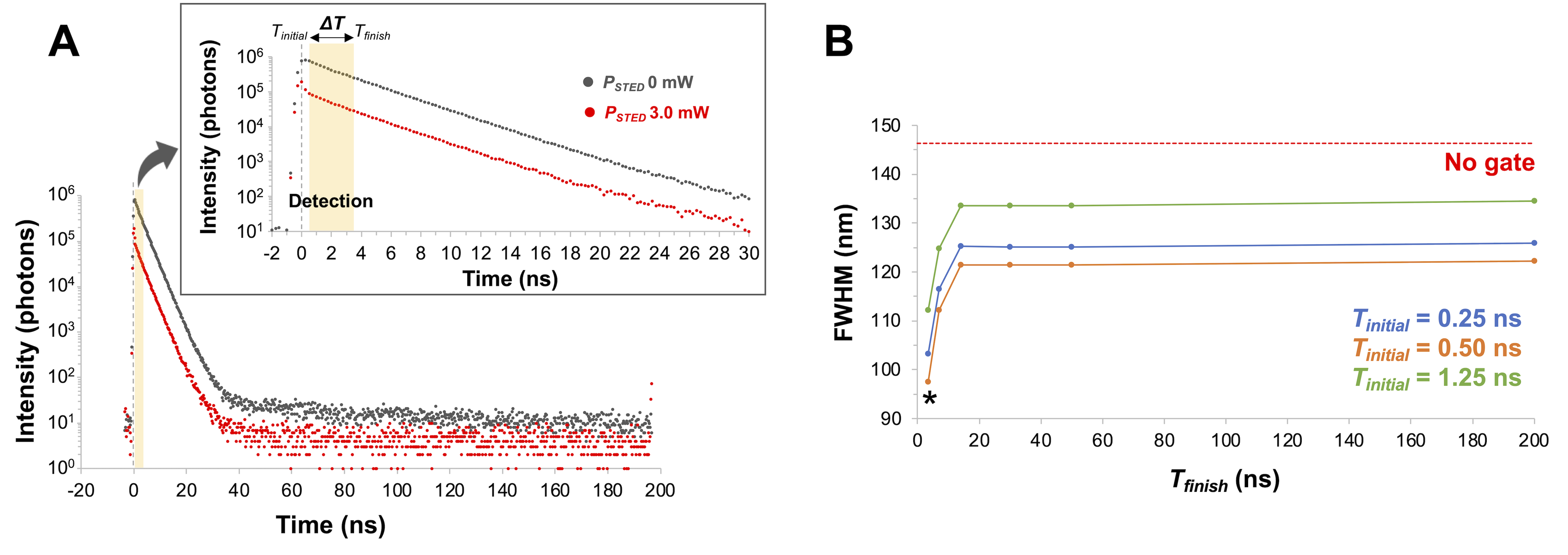

Supplement: S5 Fig — (A) Histogram of the photon-arrival times of the fluorescence of the neuronal dendrites in the immunostained fixed brain slices observed via 2PE or 2PE-STED microscopy. The constructed images are shown in Fig 4. (B) The FWHM values along the spine neck in the 2PE-STED image were evaluated within each gating window. The dotted red line indicates the FWHM value without time-gating (no gate). Tinitial was set to 0.25 ns, 0.50 ns, and 1.25 ns. Tfinish was set arbitrarily. Setting ΔT to 0.50–3.50 ns gave the best FWHM (asterisk). (TIF) [file pone.0290550.s005.tif]

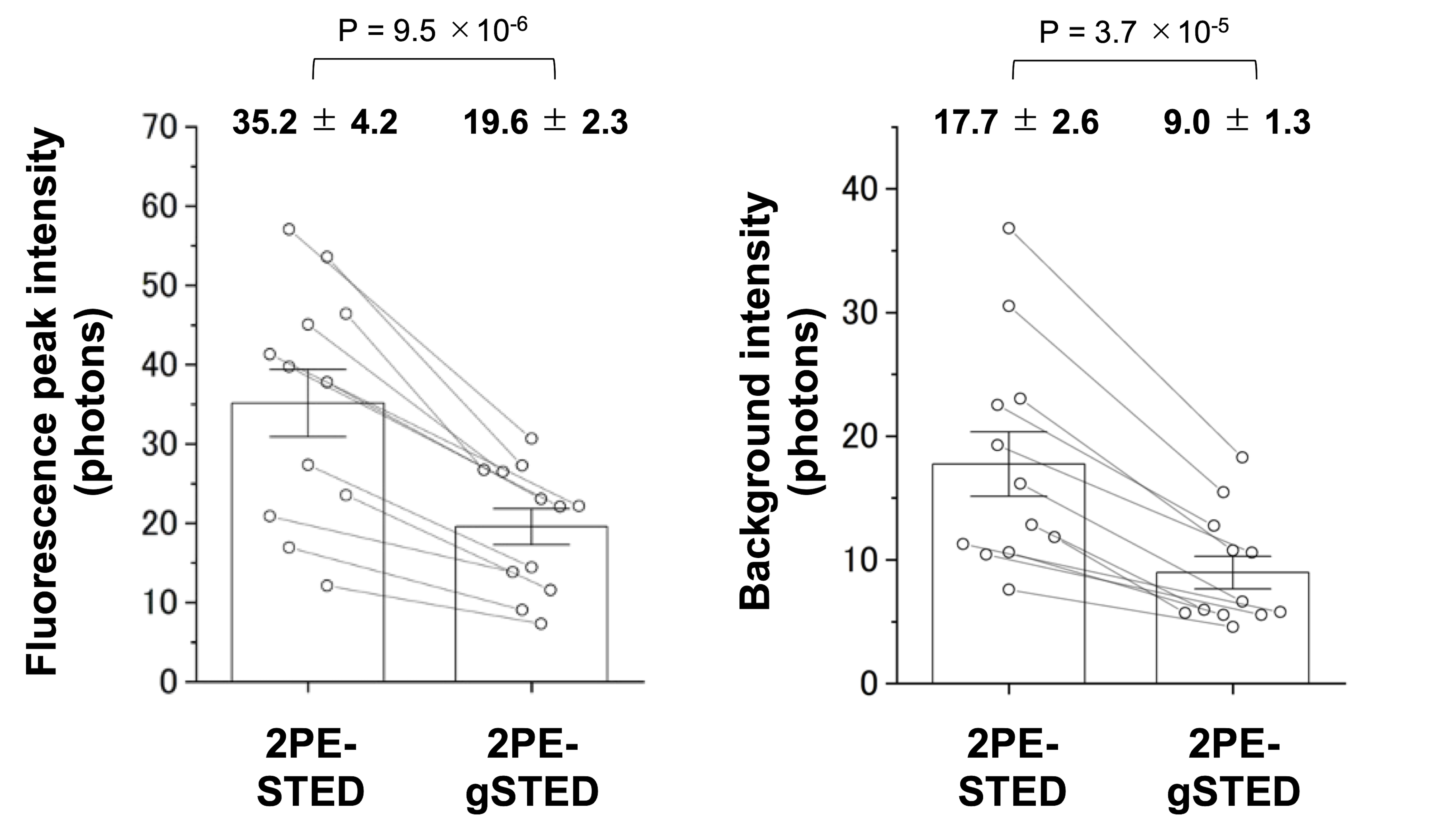

Supplement: S6 Fig — The fluorescence peak and background intensities in the local area around the spine necks in the 2PE-STED and 2PE-gSTED images in Fig 4 were obtained from the Gaussian curve parameters. The fluorescence peak intensities were obtained from the amplitude parameters, and the background intensities were obtained from the offset parameters. The error bars represent the s.e.m. The P-values are from a paired Student’s t-test. (TIF) [file pone.0290550.s006.tif]
