# Supplementary material for: All-synchronized picosecond pulses and time-gated detection improve the spatial resolution of two-photon STED microscopy in brain tissue imaging
Source: PLoS One. 2023 Aug 24;18(8):e0290550. doi: 10.1371/journal.pone.0290550 (PMC10449175; doi:10.1371/journal.pone.0290550)
Supplement: Attachment — Submitted filename: Comments to the Authors.docx [file pone.0290550.s008.docx]

Comments to the Authors

1) How to generate and adjust the time delay between 2PE and STED pulses and what is the specific value? Please describe it in detail in the article.
2) The pictures in the paper are suggested to be provided in high resolution.
3) What are the factors that affect the gate window? And how to determine the gate window, including the *T*_finish_ and *T*_initial_.
4) It is suggested to use signal-to-noise ratio (SNR) or signal-to-back ratio (SBR) to quantify the effectiveness of this method in improving image quality.
